# Diagnostic accuracy of lung ultrasound to predict weaning outcome: a systematic review and meta-analysis

**DOI:** 10.3389/fmed.2024.1486636

**Published:** 2024-11-01

**Authors:** Zhiyang Zhang, Li Guo, Huawei Wang, Ze Zhang, Limin Shen, Heling Zhao

**Affiliations:** ^1^Department of Critical Care Medicine, Hebei Medical University, Shijiazhuang, China; ^2^Department of Intensive Care Unit, Hebei General Hospital, Shijiazhuang, China; ^3^Department of Neonatal, Shijiazhuang Fourth Hospital, Shijiazhuang, China

**Keywords:** ultrasonography, extubation, weaning, critical care, meta-analysis

## Abstract

**Background:**

This systematic review and meta-analysis aim to systematically assess the diagnostic accuracy of lung ultrasound in predicting weaning failure from mechanical ventilation in critically ill patients.

**Methods:**

We searched the relevant literature up to January 2024 in the databases Web of Science, Cochrane Library, Embase, and PubMed. Two researchers independently screened eligible studies and extracted data; disagreements, if any, were resolved through discussion or consultation with a third-party expert. The quality of the included studies was assessed using the Quality Assessment of Diagnostic Accuracy Studies-2 tool. Statistical analyses were performed using Review Manager version 5.3 and Stata version 18.0, applying bivariate random-effects models to estimate sensitivity, specificity, diagnostic odds ratios, and their 95% confidence intervals, as well as to summarize receiver operating characteristic curves. Inter-study heterogeneity was assessed using the I-squared statistic, and potential sources of heterogeneity were explored by meta-regression analysis. The study follows the guidelines for Preferred Reporting Items for Systematic Reviews and Meta-Analyses in reporting.

**Results:**

Fourteen studies were included in the systematic review, of which 13 studies (totaling 988 patients) were included in the meta-analysis. The meta-analysis revealed an overall sensitivity of 0.86 (95% confidence interval: 0.77–0.91) and a specificity of 0.75 (95% confidence interval: 0.66–0.83) for lung ultrasound in predicting extubation failure. The area under the receiver operating characteristic curve was 0.87 (95% confidence interval: 0.84–0.89). Meta-regression analysis identified lung ultrasound thresholds, reference standards (extubation outcomes), and study flow and time bias as significant factors influencing diagnostic accuracy.

**Conclusion:**

This systematic review and meta-analysis demonstrated that lung ultrasound has high diagnostic accuracy in predicting extubation failure in mechanically ventilated critically ill patients. Despite some study heterogeneity, lung ultrasound proved to be a reliable predictive tool for extubation failure. Future research should focus on standardizing the definition of extubation failure, exploring the impact of different thresholds on the predictive ability of lung ultrasound, and validating its application in various clinical settings to enhance its utility and accuracy in clinical practice.

**Systematic review registration:**

This systematic review and meta-analysis was registered with PROSPERO (registration number: CRD42024555909). The study adhered to the guidelines set by the Preferred Reporting Items for Systematic Reviews and Meta-Analyses (PRISMA). Details of the PROSPERO protocol can be found in Supplementary Table 1.

## Introduction

One of the major challenges faced by the scientific community is determining the optimal timing for extubation. This involves assessing the patient’s current health and predicting potential post-extubation complications, making the decision-making process particularly complex (1). Although mechanical ventilation (MV) is widely used in intensive care units (ICUs) and saves countless lives daily, prolonged reliance on mechanical ventilation significantly increases the risk of mortality and complications (2–5). If extubation is performed prematurely, the patient may require reintubation due to failure to wean from the ventilator, exposing them to unnecessary hemodynamic and respiratory stress. Conversely, delayed extubation prolongs mechanical ventilation, increasing the risk of ventilator-associated pneumonia, tracheal injuries, and barotrauma (6–8). Thus, both premature and delayed extubation can elevate mortality, prolong ICU stays, and increase healthcare costs (9). To optimize the timing of ventilator withdrawal, healthcare professionals rely on various indices to assess a patient’s readiness for extubation. These indices include physiological measures such as minute ventilation and inspiratory pressure, along with clinical indicators like the rapid shallow breathing index and tracheal obstruction pressure. This comprehensive approach helps to navigate the complexities involved in extubation decision-making. However, despite these methods, studies have shown that approximately 20% of patients still experience challenges during weaning and extubation (10, 11).

Ultrasound technology has proven to be a highly reliable and accurate tool for monitoring internal organ abnormalities, often surpassing traditional radiological methods (12–14). In particular, ultrasound assessments of diaphragm function, such as diaphragm excursion (DE) and diaphragm thickening fraction (DTF), have been shown to predict weaning success (15, 16). Meta-analyses also support their role in predicting weaning failure (17–20). A study by Llamas-Álvarez et al. (19) suggested that the lung ultrasound score (LUS) may serve as a valid predictor of extubation outcomes, although the study included only five trials, and the results were less conclusive.

Lung ultrasound (LUS) scoring, as initially described by Bouhemad et al. (21), quantitatively assesses pulmonary ventilation by examining different lung regions bilaterally, determining whether lung ventilation is adequate or compromised. This method has been clinically applied to monitor the pulmonary status of mechanically ventilated patients, guiding decisions regarding readiness for weaning. Over time, the use of LUS has enabled clinicians to track changes in lung conditions, aiding in informed decision-making about the continuation or cessation of mechanical support. Despite its potential, variability in LUS score thresholds across studies—used to determine successful spontaneous breathing trials (SBT) and extubation—has led to inconsistent conclusions. Therefore, this systematic review and meta-analysis aims to delve deeper into the diagnostic accuracy of lung ultrasound in predicting extubation failure among mechanically ventilated critically ill patients, providing more precise and practical insights for clinical practice.

## Methods

### Study protocol

This systematic review and meta-analysis aimed to evaluate the diagnostic accuracy of the lung ultrasound score (LUS) in predicting extubation failure among mechanically ventilated, critically ill patients. Before initiating the study, a detailed protocol was developed and registered with PROSPERO (registration number: CRD42024555909) (see Supplementary Table 1 for the PROSPERO Protocol). The study adhered strictly to the guidelines set by the Preferred Reporting Items for Systematic Reviews and Meta-Analyses (PRISMA) (22).

### Literature search and selection

Two independent reviewers conducted comprehensive literature searches in databases, including Web of Science, Cochrane Library, Embase, and PubMed, up until January 7, 2024. To ensure thorough coverage, references from relevant studies were also examined, without imposing restrictions on study design or language. EndNote software was used for managing and screening the literature. The search process continued until no additional relevant studies were found. Search terms included Medical Subject Headings (MeSH) and other standardized terms, and a concept-based approach was employed to capture terms related to “extubation,” “weaning,” “ultrasound,” “ultrasonography,” and “echography.” The complete search strategy is available in Supplementary Table 2. In cases of disagreement between reviewers, a third researcher was consulted to reach a consensus.

### Inclusion and exclusion criteria

Studies were included if they met the following criteria: (1) critically ill adult patients (aged ≥18 years) on mechanical ventilation in the ICU who were deemed ready for withdrawal, and (2) patients who had undergone lung ultrasonography with complete data on withdrawal outcomes. Extubation failure was defined as the need for reintubation (invasive or noninvasive mechanical ventilation), tracheostomy, or death within 72 h of withdrawal, or failure of the spontaneous breathing trial (SBT). Success of withdrawal was defined as the absence of extubation failure criteria. Exclusion criteria were: (1) duplicate or overlapping studies, (2) studies lacking sufficient data to construct a 2 × 2 table, and (3) unpublished data.

### Rationale for excluding patients with noninvasive ventilation post-extubation

Patients who received noninvasive ventilation post-extubation were excluded, as these patients might require reintubation, which could confound the assessment of extubation outcomes. While noninvasive ventilation is commonly used to enhance lung recruitment and reduce respiratory muscle workload, its use could obscure the true outcomes of weaning failure. This exclusion was intended to reduce variability and ensure that our study results accurately reflect the diagnostic accuracy of lung ultrasound scores in predicting extubation failure. We acknowledge that this decision may limit the generalizability of our findings but believe it is essential for maintaining scientific rigor and ensuring the practical utility of the results.

### Data extraction

Two researchers independently extracted data from the included studies, with a third researcher resolving any disagreements. The extracted data included the study ID (first author and year of publication), country, study design, setting, inclusion and exclusion criteria, ultrasound probe frequency, lung regions examined, the scoring system used, critical score values, methods for spontaneous breathing trials, and ultrasound parameters predicting extubation failure (categorized as true positives, true negatives, false positives, and false negatives).

### Quality assessment and publication bias

The methodological quality of each study was evaluated using the Quality Assessment of Diagnostic Accuracy Studies-2 (QUADAS-2) tool (23), which assesses four areas of bias: patient selection, index tests, reference standards, and process and timing. Publication bias was assessed using funnel plots, and potential bias was determined by examining graphical asymmetry (24).

### Statistical analysis

Statistical analyses were conducted using Review Manager 5.3 and Stata 18.0 software, utilizing the “metandi” function (25). Bivariate meta-analysis models (26) were applied to estimate sensitivity, specificity, positive and negative likelihood ratios, and diagnostic odds ratios, with corresponding 95% confidence intervals calculated. Summary receiver operating characteristic (SROC) curves and their areas under the curve (AUC) were generated to evaluate the accuracy of lung ultrasound scores in predicting extubation failure (27). The closer the SROC curve is to the upper left corner, the larger the AUC, indicating higher overall test accuracy. Non-threshold heterogeneity was assessed using the *Q*-value and *I*^2^ statistic (28). Fagan plots were applied to assess the clinical applicability of lung ultrasound in diagnosing weaning outcomes.

### Meta-regression analysis

Meta-regression analyses were performed to explore study characteristics associated with the diagnostic accuracy of diaphragmatic ultrasound and to identify potential sources of heterogeneity in sensitivity and specificity. These characteristics included the cut-off value of the lung ultrasound score (LUS), index test, reference standard, process and timing, patient selection, and study quality.

## Results

### Study identification and selection

**Figure 1:**
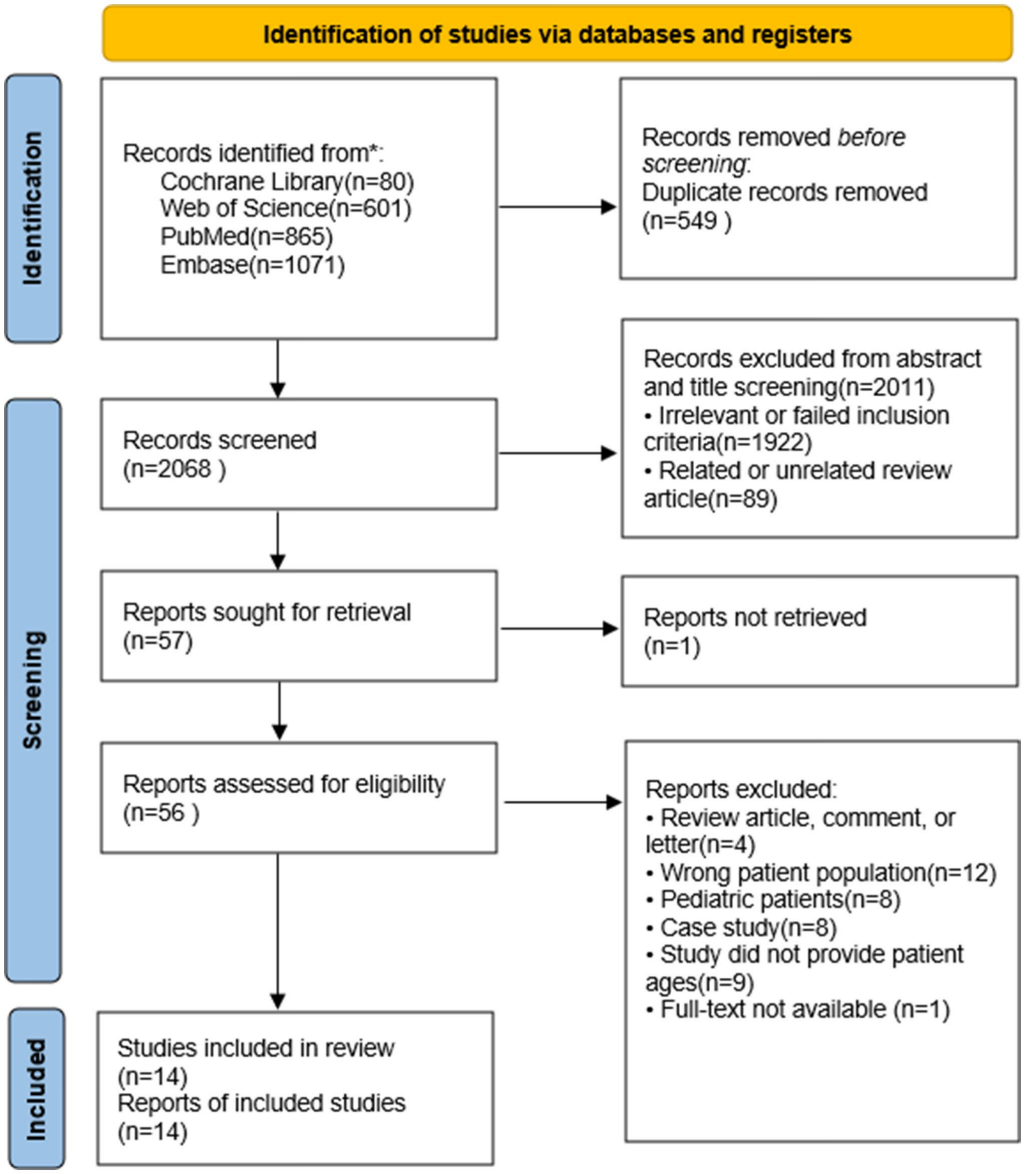
Flow diagram of the literature and selection in the meta-analysis.

A detailed flowchart of the literature search is shown in Figure 1. The initial database search yielded 2,617 references. After the removal of duplicates, 2,068 records remained. Screening of titles and abstracts resulted in 57 studies, of which 43 were excluded after full-text review (see Figure 1 for details on exclusion reasons). Ultimately, 14 studies published between 2012 and 2023 were included in the analysis (29–42); one study was included in the qualitative analysis (42), and the remaining 13 were included in the quantitative analysis, evaluating 988 patients for lung ultrasound scores.

### Baseline characteristics of included studies

The baseline characteristics of the included studies are presented in Tables 1, 2; Supplementary Table 3. All the studies were prospective cohort studies. Among the 14 studies, 4 were conducted in Egypt (29, 31, 34, 41), 2 in France (30, 39), 2 in the United States (33, 35), 2 in China (38, 40), and the remaining 4 in Spain (36), Brazil (42), Nepal (37), and India (32).

**Table 1:** Characteristics of included studies.

Study/year	Country	Design	Definition of weaning failure	Ultrasound probe frequency
Osman et al./2017	Egypt	Prospective observational	Reintubation within 48 h	3.5 MHz
Binet et al./2014	France	Prospective observational	Reintubation, nonscheduled NIMV, or death within 48 h	NR
Soliman et al./2019	Egypt	Prospective observational	Failed SBT or the need for reintubation within 48 h following extubation	2–4 MHz
Banerjee et al./2018	India	Prospective observational	Reintubation within 48 h	NR
Gok et al./2021	America	Prospective observational	Reintubated within 48 h, need for noninvasive mechanical ventilation	2–4 MHz
Shoaeir et al./2016	Egypt	Prospective observational	Need for reintubation and mechanical ventilation, need for noninvasive positive pressure ventilation	2–4 MHz
Gosai et al./2021	America	Prospective observational	Reintubation and mechanical ventilation after 48 h	3.5–6.5 MHz
Tenza-Lozano et al./2018	Spain	Prospective observational	The occurrence of reintubation, non-invasive ventilatory support or death within 48 h following extubation.	2–4 MHz
Antonio et al./2018	Brazil	Prospective observational	An inability to tolerate a T-piece SBT of 30–120 min	2–4 MHz
Rajbanshi et al./2023	Nepal	Prospective observational	Require any form of invasive or noninvasive respiratory support within 48 h of extubation	2–4 MHz
Wang et al./2022	China	Prospective observational	Failed SBT or the need for reintubation within 48 h following extubation	3.5 MHz
Soummer et al./2012	France	Prospective observational	Ventilatory support (either noninvasive or invasive ventilation) within 48 h after extubation	2–4 MHz
Xu et al./2020	China	Prospective observational	The requirement for NPPV or reintubation within 48 h	NR
Gu et al./2020	Egypt	Prospective observational	Reintubation and mechanical ventilation after 48 h	2–4 MHz

NIMV, noninvasive mechanical ventilation; SBT, spontaneous breathing trial; NPPV, noninvasive positive-pressure ventilation.

**Table 2:** Participant characteristics.

Study	Patients	Age^a^	Setting	Inclusion	Type of SBT
Osman et al.	68	56 (45–65)	MICU SICU	Patients ready for SBT	T-tube
Binet et al.	48	59 ± 16	MD ICU	Ventilated >48 h	PS
Soliman et al.	100	57 ± 14	ICU	Ventilated >48 h	PS
Banerjee et al.	53	Female 55.52 ± 6.39 Male 55.07 ± 5.2	ICU	Patients ready for SBT	T-tube/PS
Gok et al.	62	57.6 ± 14.1	ICU	Ventilated >48 h	T-tube/PS
Shoaeir et al.	50	SG 47.52 ± 14.60 FG 51.89 ± 14.58	ICU	Patients ventilated >48 h and ready for a first SBT	NR
Gosai et al.	100	62 (26–72)	EICU	Patients ready for SBT	NR
Tenza-Lozano et al.	69	66 (53, 78)	ICU	Ventilated >24 h; ready for weaning	T-tube/PS
Antonio et al.	250	SG 75 (60–83) FG 66 (47–81)	MICU SICU	Undergone invasive mechanical ventilation for 24 h	NR
Rajbanshi et al.	102	43.52 ± 19.50	MICU SICU	Mechanically ventilated for a duration exceeding 24 h and were considered ready for the weaning process	NR
Wang et al.	92	SG 60.25 ± 13.64 FG 66.92 ± 14.53	ICU	Patients who met the diagnostic standard of ARDS (the Berlin definition). Patients ready for SBT	PS
Soummer et al.	86	60 ± 15	2 MD ICU	Patients ventilated >48 h who successfully passed a first SBT	T-tube
Xu et al.	105	57.98 ± 15.20	ICU	Successful SBT and considered ready for extubation were recruited	NR
Gu et al.	53	68 (53–77)	ICU	Acute moderate and severe respiratory failure, Patients ventilated >48 h and ready for a first SBT	T-tube

ICU, intensive care unit; MICU, medical intensive care unit; SICU, surgical intensive care unit; MD, multidisciplinary; EICU, emergency intensive care unit; SG, success group; FG, failure group; SBT, spontaneous breathing trial; ARDS, acute respiratory distress syndrome; PS, low pressure support trial, NR, not reported.

a Age is expressed according to data extracted from each study as mean ± standard deviation or median (interquartile range).

Most of the studies were conducted in general ICUs, but three were carried out in medical and surgical ICUs (29, 36, 37), and one in an emergency ICU (35). Thirteen studies performed a quantitative assessment of lung ultrasound and provided scores, while one study used a qualitative assessment (42). Ten of the quantitative studies restricted the scans to the anterior, posterior, and lateral lung regions, defining the lung ultrasound score range as 0–36. Three additional studies restricted the scans to the anterior and lateral lung regions, with a lung ultrasound score range of 0–24 (33, 35, 36). Further details regarding lung ultrasound score definitions and LUS cut-offs for each study can be found in Supplementary Table 3.

The definition of “failure to extubate” varied among the studies and included one or more of the following: inability to maintain spontaneous respiration without ventilatory support for more than 48 h, the need for noninvasive ventilation, high-flow nasal cannula, reintubation, terminal extubation, tracheostomy, delayed extubation, and/or failure of the spontaneous breathing trial (SBT). Patient inclusion criteria and modes of extubation also differed across studies, as detailed in Tables 1, 2.

### Assessment of methodologic quality

**Figure 2:**
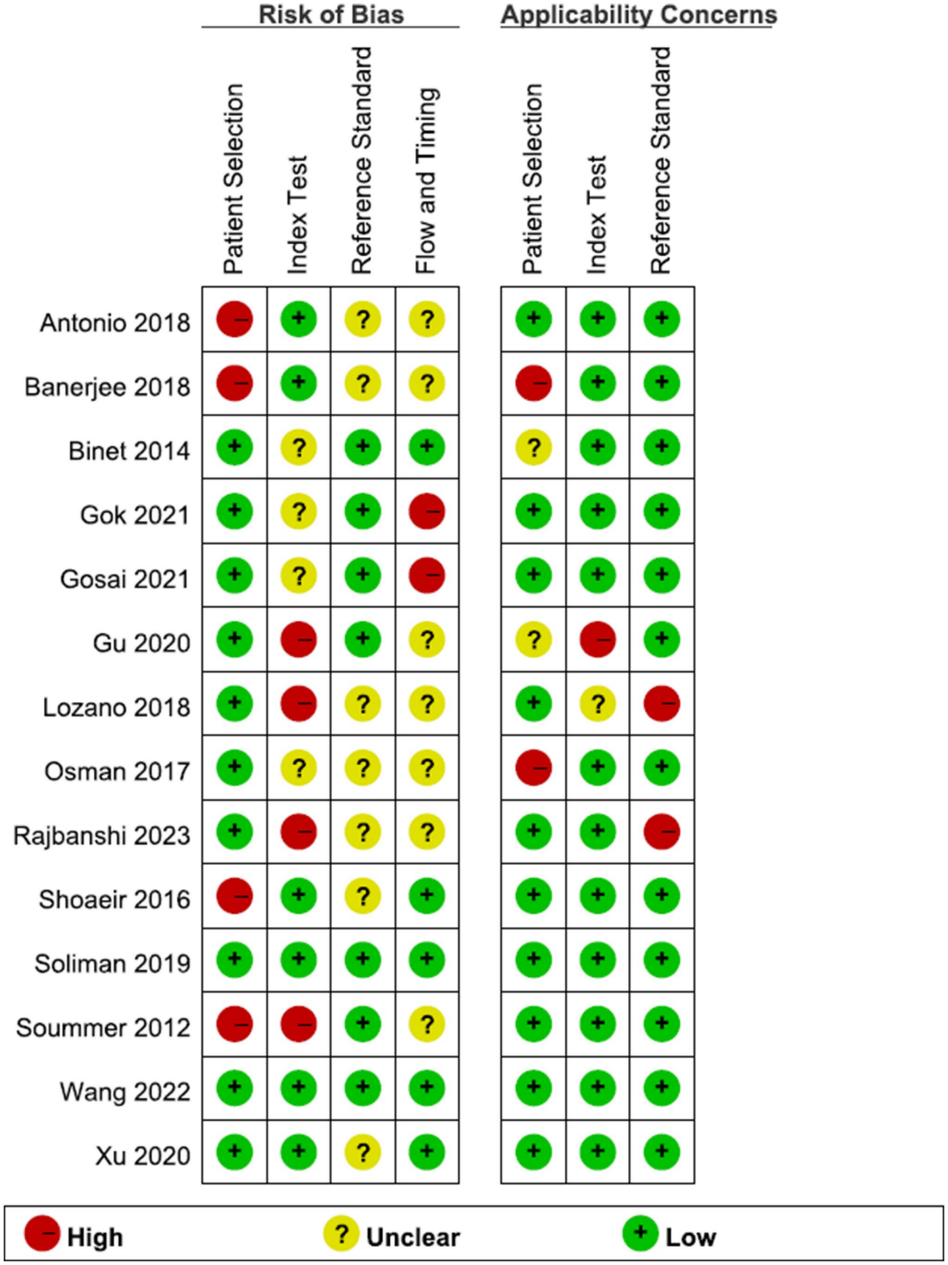
Risk of bias and applicability concerns assessment with the Quality Assessment of Diagnostic Accuracy Studies (QUADAS).

The results of the Cochrane risk of bias assessment for the studies included in this systematic review are shown in Figure 2. Most studies demonstrated an unclear or high risk of bias regarding the index test and reference standards. The majority of the studies [except three (32, 39, 40)] did not report whether the lung ultrasound (index test) was interpreted independently of the extubation outcomes. Nearly half of the studies did not disclose the identity of the ultrasound operators. Lung ultrasounds were typically performed before or during the spontaneous breathing trials (SBT), but two studies (39, 40) only considered individuals who successfully passed these tests (see Table 2). Most studies [except for three (30, 39, 40)] provided varying definitions of extubation outcomes (reference standard), and these definitions were not always adequately described. One study defined extubation failure solely as a failure in the spontaneous breathing trial, which presented a high risk of bias.

### Diagnostic accuracy and heterogeneity

The meta-analysis included 13 studies, excluding one study due to its qualitative design, which involved a different range of ultrasound scans and lacked a quantitative score.

**Figure 3:**
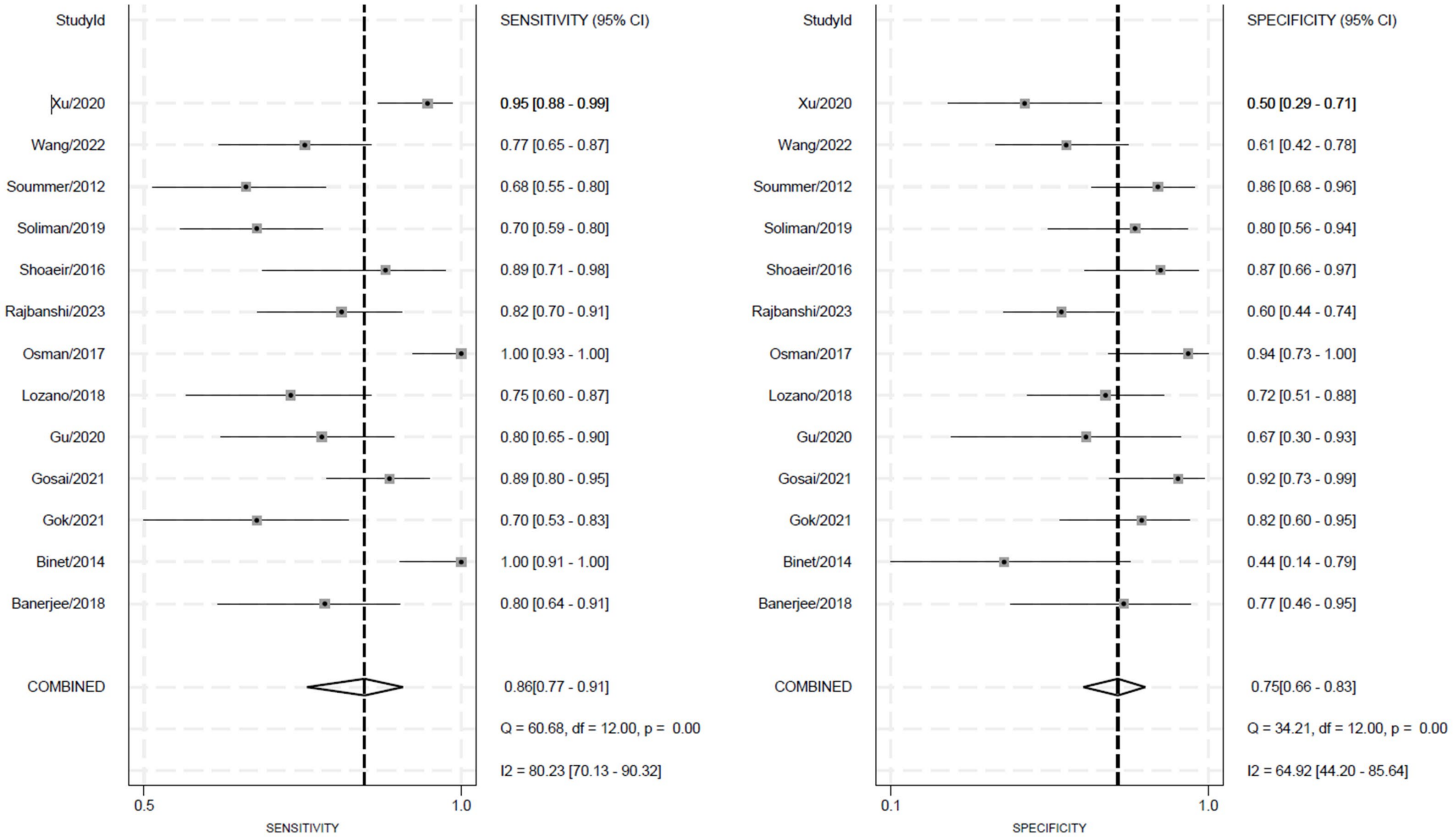
Forest plot of sensitivity and specificity.

**Figure 4:**
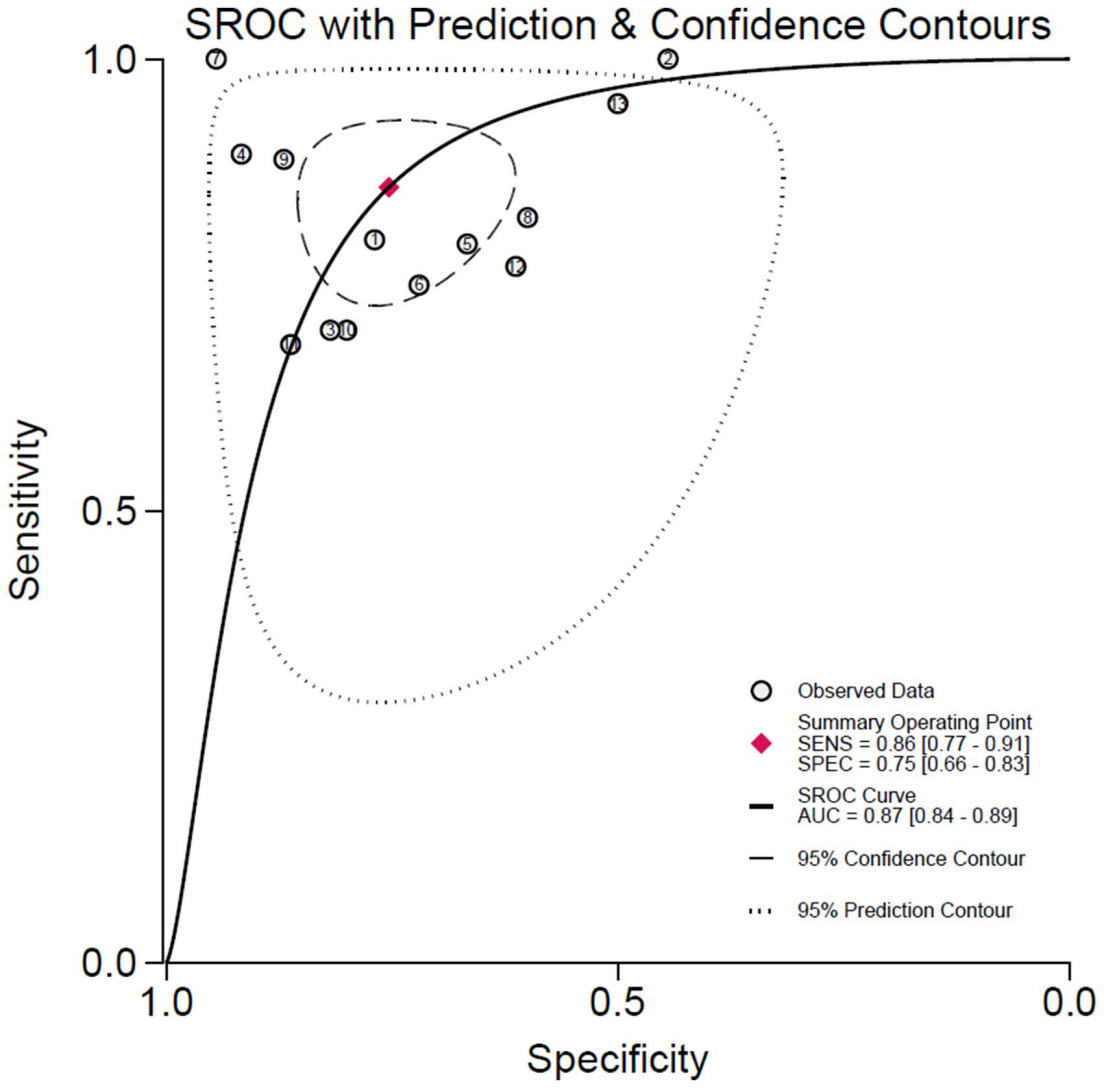
Summary receiver-operating characteristic curve illustrating the summary point and the study estimate of the sensitivity and specificity, and the 95% confidence and prediction contour for weaning failure prediction.

Figure 3 displays the forest plots for the sensitivity and specificity of ultrasound scores across the included studies. Figure 4 shows the summary receiver operating characteristic (SROC) curves, illustrating the pooled points and 95% confidence intervals for the sensitivity and specificity of the lung ultrasound score (LUS) (AUC = 0.87, 95% CI 0.84–0.89). The pooled sensitivity and specificity for LUS were 0.86 (95% CI 0.77–0.91) and 0.75 (95% CI 0.66–0.83), respectively. Pooled diagnostic odds ratios and likelihood ratios are presented in Supplementary Figure 1. Heterogeneity in sensitivity and specificity for LUS was significant, with the *Q*-test = 60.68, *p* < 0.01, *I*^2^ = 80.23% for sensitivity, and *Q*-test = 34.21, *p* < 0.01, *I*^2^ = 64.92% for specificity.

Fagan plots were used to demonstrate the shift in probabilities of extubation failure based on lung ultrasound outcomes (Supplementary Figure 2). The initial pre-test probability was set at 50%, representing the median risk in similar clinical populations and typical uncertainties in extubation decisions. For patients with a positive LUS result (exceeding the cut-off value), the post-test probability of extubation failure increased to 78%, indicating a high likelihood of needing continued mechanical support. Conversely, a negative LUS result (below the cut-off) reduced the post-test probability to 16%, suggesting a lower risk and potentially safer conditions for extubation. These probability adjustments were based on likelihood ratios derived from the sensitivity and specificity values of LUS in predicting extubation failure.

### Meta-regression analysis

The results of the meta-regression analysis for LUS sensitivity and specificity are shown in Supplementary Figures 3–6. Factors such as thresholds, risk of bias in article quality, reference standards, and study flow and timing, as identified in the QUADAS-2 assessment, were analyzed as potential sources of heterogeneity in the diagnostic accuracy of lung ultrasound. A threshold of >15 points significantly increased the sensitivity of LUS in predicting extubation failure (*p* = 0.02). Studies with low risk in QUADAS-2 domains were categorized as high quality. However, there was no significant difference in diagnostic accuracy between high-and low-quality studies. A low risk of bias in the reference standard (extubation outcomes) significantly increased the sensitivity of LUS (*p* = 0.01), while a low risk of bias in patient flow and timing significantly increased the specificity of LUS (*p* = 0.01).

### Sensitivity analysis and publication bias

Sensitivity analysis, including impact analysis and outlier detection, identified two outliers for LUS (see Supplementary Figure 7). Excluding these outliers in subgroup analysis resulted in a slightly reduced sensitivity (0.81, 95% CI 0.74–0.86) and an almost unchanged specificity (0.75, 95% CI 0.66–0.82). The fitness and bivariate normality analyses confirmed the moderate robustness of the random-effects bivariate model used in this meta-analysis.

The Deeks funnel plot test did not detect significant publication bias for LUS (see Supplementary Figure 8).

## Discussion

This systematic review and meta-analysis aimed to assess the diagnostic accuracy of lung ultrasound (LUS) scoring in predicting extubation failure in mechanically ventilated, critically ill patients. The LUS score has recently gained recognition as a simple and reliable method, particularly for diagnosing lung disease in the intensive care unit (ICU) setting (43). LUS provides a wealth of information about morphological changes in the lungs, aiding physicians in assessing the progression of lung disease by observing ultrasound signs such as abnormal pleural lines, increased B-lines, and solid lung lesions. The total LUS score in this study reflects the severity of ventilation loss in both lungs (44).

The LUS score, known for integrating respiratory and cardiac assessments, functions as a composite tool rather than merely a diagnostic indicator, offering a comprehensive view of patient status. This dual utility is especially crucial when diastolic dysfunction alters LUS scores post-extubation, transitioning from a low to high score. These findings underscore the importance of nuanced interpretation of LUS score changes over time.

Our results support the utility of LUS as a valuable predictor of extubation failure, with a pooled sensitivity of 0.86, specificity of 0.75, and an area under the receiver operating characteristic (ROC) curve (AUC) of 0.87, indicating high reliability. These results align with those from Mohsen’s et al. (45) neonatal systematic evaluation (AUC of 0.87) and are consistent with findings by Biasucci et al. (46), who also noted that LUS is an accurate and useful diagnostic tool across critically ill patients of all ages. These consistent findings reinforce the value of LUS in predicting extubation failure in mechanically ventilated, critically ill patients.

Banerjee et al. (32) reported that patients with LUS scores between 1 and 10 had a high rate of successful extubation, while those with scores between 16 and 32 had a higher rate of extubation failure. This is consistent with our meta-regression analysis, which showed that using a threshold greater than 15 points significantly increased the sensitivity of LUS in predicting extubation failure. These results highlight the importance of selecting appropriate LUS thresholds in clinical practice, aligning with findings from Amara et al. (47), who observed significant differences in LUS between simple and complex extubation scenarios, further demonstrating the utility of LUS in nuanced clinical assessments.

Additionally, the rapid acquisition and ease of use of LUS make it a time-efficient tool that, when used alongside other clinical assessments, significantly enhances the accuracy and speed of decision-making. This efficiency is particularly valuable in high-pressure ICU environments, such as those managing COVID-19 patients. For example, Meroi et al. (48) demonstrated that in COVID-19 ICUs, LUS allowed for significant time savings compared to traditional radiology, with assessments taking approximately 4.2 min per patient. This rapid evaluation enables timely monitoring of pulmonary conditions, minimizing exposure to healthcare workers and conserving critical resources. Furthermore, extubation failure is not always related to lung pathology but can also result from upper airway issues, such as laryngeal edema, which may require additional evaluation methods like the leak test. Moreover, LUS has been shown to reduce healthcare costs by decreasing the need for patient transport to radiology for chest CT scans and reducing X-ray exposure (49). The cost savings from fewer diagnostic imaging procedures and complications during patient transfers position LUS as a cost-effective alternative in respiratory care.

Previous systematic reviews (17) have noted that inconsistencies in defining extubation failure across studies may affect the accuracy of diaphragm ultrasound in predicting extubation outcomes. This issue also applies to using LUS for predicting extubation failure. Despite focusing on different anatomical areas, both diaphragm ultrasound and LUS have potential value in assessing readiness for extubation. Therefore, our findings emphasize the need for strict and consistent criteria when using LUS to predict extubation failure. Standardizing definitions of extubation failure will improve the sensitivity and specificity of predictions, suggesting that a uniform and standardized definition is essential for improving prediction accuracy in both diaphragmatic ultrasound and LUS-based assessments.

Our study also found that reducing bias in patient flow and timing significantly increased the specificity of LUS. This underscores the importance of reducing bias in study design and implementation to improve the accuracy of LUS in predicting successful extubation. Variations in patient inclusion and exclusion criteria, as well as inconsistencies in ultrasound operators and operation times, introduced potential biases that may have affected the specificity of LUS. Thus, conducting rigorous and standardized studies is crucial to ensuring the accuracy and reliability of LUS scores in predicting extubation failure. This includes clearly defining patient inclusion and exclusion criteria, standardizing ultrasound procedures, and controlling for potential confounders during analysis. By adhering to rigorously designed studies, we can ensure the validity and reliability of LUS scores in predicting extubation failure in mechanically ventilated, critically ill patients, ultimately improving patient outcomes.

### Strengths and limitations

In recent years, significant attention has been given to the diagnostic accuracy of lung ultrasound (LUS) scores in predicting extubation failure in mechanically ventilated, critically ill patients. Compared to earlier studies from Llamas-Álvarez et al. (19), this systematic review added nine new relevant studies, expanding the meta-analysis to include more uniform and clinically relevant data. This enhancement increases the reliability and representativeness of the results. Additionally, we thoroughly explored the sources of sensitivity and specificity heterogeneity in LUS scores. Through meta-regression analyses, we identified potential factors influencing the accuracy of LUS, providing valuable guidance for its application in different clinical settings and future studies.

However, there are some limitations to this systematic review and meta-analysis. First, unpublished data were excluded to maintain study quality, which may have resulted in the omission of relevant studies, potentially impacting the completeness of the findings. Despite our in-depth analysis of heterogeneity, significant differences remained between studies regarding patient populations, definitions of extubation failure, and the application of ultrasound scoring systems. These differences could affect the consistency and generalizability of the results. Moreover, some studies did not specify the identity of the ultrasound operators, which could introduce operator bias and influence the accuracy and reliability of LUS scoring.

Despite these limitations, our analysis indicates that the LUS score is a valid tool for predicting extubation failure in critically ill patients on mechanical ventilation. With further research and standardization of methods, the LUS score has the potential to become an important auxiliary diagnostic tool in clinical practice. Future research should focus on improving study design and implementation, as well as standardizing the definition of extubation failure, to enhance the accuracy and reliability of LUS in predicting extubation outcomes. Additionally, studies should explore the impact of different cut-off values on the predictive ability of LUS scores and validate its application in various clinical settings.

## Data Availability

Publicly available datasets were analyzed in this study. This data can be found here: this study is a meta-analysis, and the data analyzed were derived from previously published literature. Detailed references are provided within the article, and there are no direct links to unpublished original datasets involved.
