# Supplementary material for: Diagnostic accuracy of lung ultrasound to predict weaning outcome: a systematic review and meta-analysis
Source: Front Med (Lausanne). 2024 Nov 1;11:1486636. doi: 10.3389/fmed.2024.1486636 (PMC11563988; doi:10.3389/fmed.2024.1486636)

Supplementary Material

Supplementary Table 3.Participant's lung ultrasound characteristics

| Study | Scanned lung zones bilaterally | Total LUS and range per zone | Studies' scoring method | LUS cut‐off |
| --- | --- | --- | --- | --- |
| Ahmed M et al | 12 zones (anterior, lateral, and posterior) | 36（0-3） | 0 point Horizontal A-line (no more than two B-line) 1 point Multiple B-line either regularly spaced or irregularly spaced 2 points Multiple coalescent B-lines 3 points Complete loss of aeration Lung consolidation | LUS score≥12 |
| Binet et al | 12 zones (anterior, lateral, and posterior) | 36（0-3） | 0 point A-lines, lung sliding, two or fewer than B lines 1 point Three or more than B-lines 2 points Multiple coalescences of B-lines 3 points Pulmonary consolidation or presence of tissue pattern | LUS score≥15 |
| Soliman et al | 12 zones (anterior, lateral, and posterior) | 36（0-3） | 0 point Presence of lung sliding with A-lines or less than two isolated B lines 1 point Multiple B lines 2 points Multiple fused B lines 3 points The presence of a dynamic air bronchograms and tissue pattern | LUS score＞15.5 |
| Banerjee et al | 12 zones (anterior, lateral, and posterior) | 36（0-3） | 0 point Normal B lines ≤2 1 point Moderate loss Multiple B lies regular/irregular 2 points Severe loss Multiple coalescent B lines 3 points Complete Lung consolidation | LUS score≥16 |

| Study | Scanned lung zones bilaterally | Total LUS and range per zone | Studies' scoring method | LUS cut‐off |
| --- | --- | --- | --- | --- |
| Funda Gok et al | 8 zones(anterior and lateral ) | 24（0-3） | 0 point A-lines, lung sliding, two or fewer than B lines 1 point Three or more than B-lines 2 points Multiple coalescences of B-lines 3 points Pulmonary consolidation or presence of tissue pattern | LUS score＞6.5 |
| Shoaeir et al | 12 zones (anterior, lateral, and posterior) | 36（0-3） | 0 point Presence of lung sliding with A lines or fewer than two isolated B lines. 1 point Multiple well-defined Blines (B1 lines). 2 points Multiple coalescence B-lines (B2 lines) 3 points The presence of a tissue pattern characterized by dynamic air bronchograms | LUS score＞18 |
| Jigarkumar B et al | 8 zones(anterior and lateral ) | 24（0-3） | 0 point Presence of lung sliding A line Fewer than two isolated b lines 1 point More than 2 well defined B-lines 2 points Multiple coalescing B lines 3 points Presence of tissue pattern | LUS score＞8.14 |
| Lozano et al | 8 zones(anterior and lateral ) | 24（0-3） | 0 point Presence of lung sliding with A lines and fewer than two isolated B lines 1 point More than two welldefined B lines 2 points Multiple coalescing B lines 3 points Presence of a tissue pattern | LUS score＞7 |

LUS: lung ultrasound

| Study | Scanned lung zones bilaterally | Total LUS and range per zone | Studies' scoring method | LUS cut‐off |
| --- | --- | --- | --- | --- |
| Antonio et al | 4 zones(between the third and fourth ribs and between the sixth and seventh ribs) | Normal and abnormal lung pattern | Interstitial syndrome: any profile with anterior bilateral B-pattern. | Abnormal pattern |
| Rajbanshi et al | 12 zones (anterior, lateral, and posterior) | 36（0-3） | 0 point Indicates normal aeration 1 point Originating from the pleural line or small consolidations near the pleura 2 points Multiple coalescent B lines are observed inseveral intercostal spaces and appear indistinct 3 points The presence of tissue echogenicity accompanied by a static or dynamic air bronchogram | LUS score＞17.5 |
| Wang et al | 12 zones (anterior, lateral, and posterior) | 36（0-3） | 0 point Lung sliding sign with A-line or less than 2 separate B-lines 1 point Multiple, typical B-line 2 points Multiple fusion B line 3 points Tissue image with typical bronchial inflation. | LUS score＞18 |
| Soummer et al | 12 zones (anterior, lateral, and posterior) | 36（0-3） | 0 point Presence of lung sliding with A lines or fewer than two isolated B lines 1 point Multiple, well-defined B lines 2 points Multiple coalescent B lines 3 points The presence of a tissue pattern characterized by dynamic air bronchograms. | LUS score≥13 |
| Study | Scanned lung zones bilaterally | Total LUS and range per zone | Studies' scoring method | LUS cut‐off |
| Xu et al | 12 zones (anterior, lateral, and posterior) | 36（0-3） | 0 point Normal aeration 1 point Multiple, well-defined B lines 2 points Multiple coalescent B lines 3 points Lung consolidation | LUS score＞15 |
| Gu et al | 12 zones (anterior, lateral, and posterior) | 36（0-3） | 0 point Normal aeration 1 point Three or more than B-lines 2 points Multiple coalescences of B-lines 3 points Pulmonary consolidation or presence of tissue pattern | LUS score＞13.5 |

Supplementary Figure 1. Forest plot of pooled diagnostic ratio ratios and pooled likelihood ratios


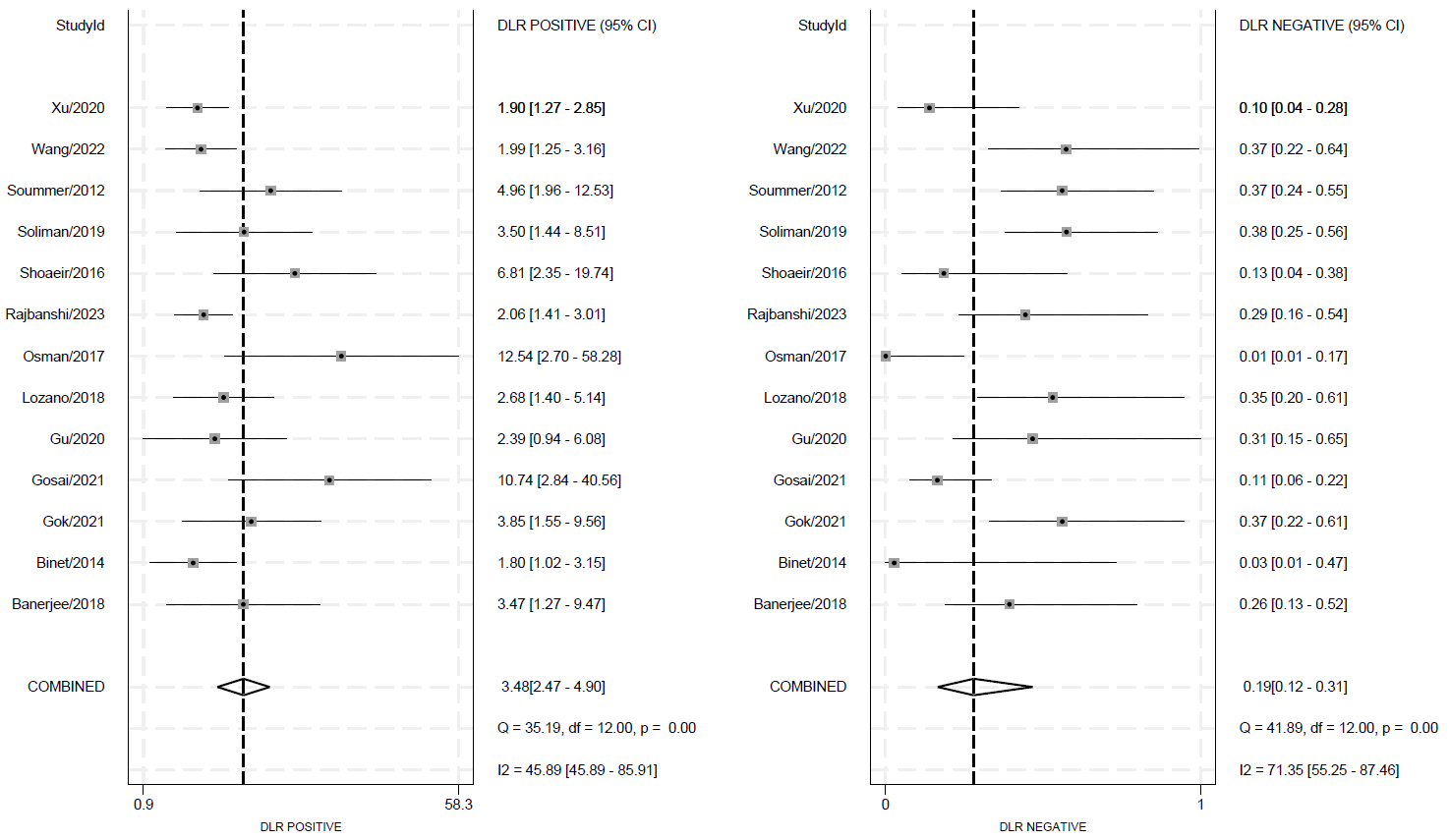


Supplementary Figure 2. Fagan Nomogram for LUS Prediction of Extubation Failure


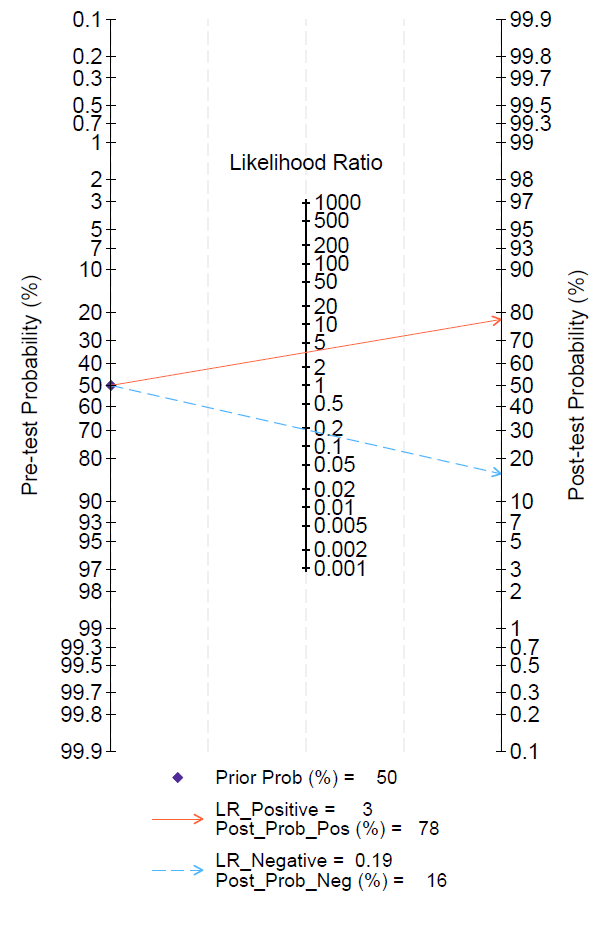


Supplementary Figure 3. Univariable Meta-regression and Subgroup Analysis of Diagnostic Sensitivity and Specificity by Reference Standard and Timing in LUS Studies


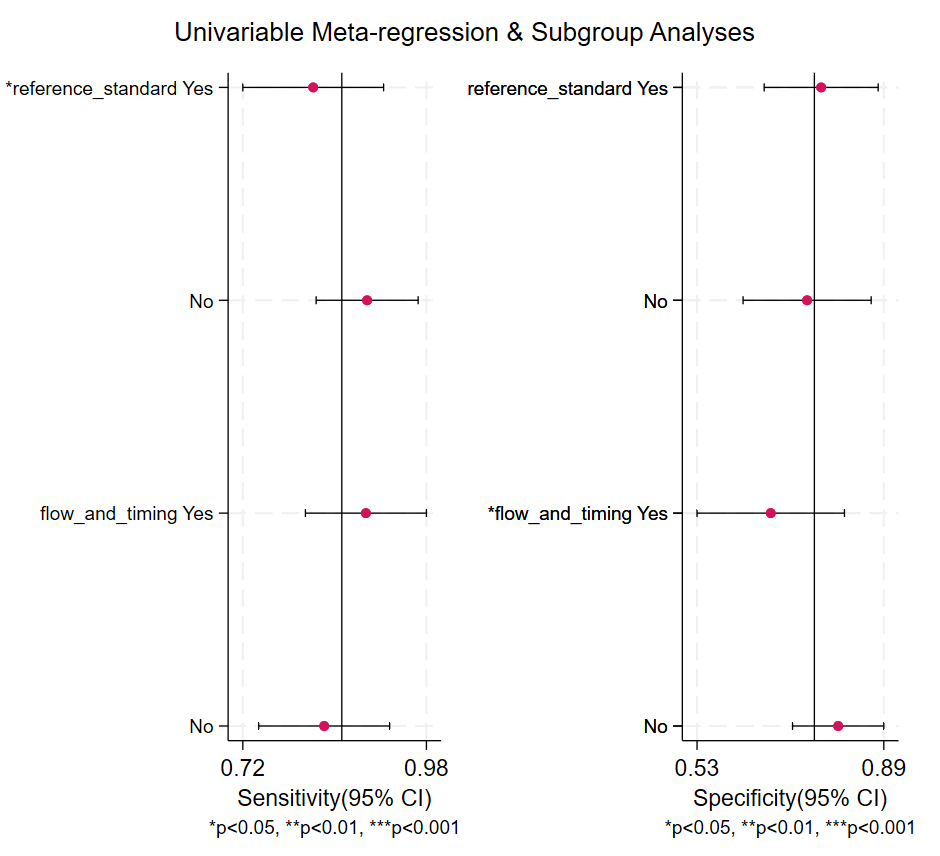


Supplementary Figure 4: Univariable Meta-regression and Subgroup Analysis of Diagnostic Sensitivity and Specificity by LUS Cut-off Values


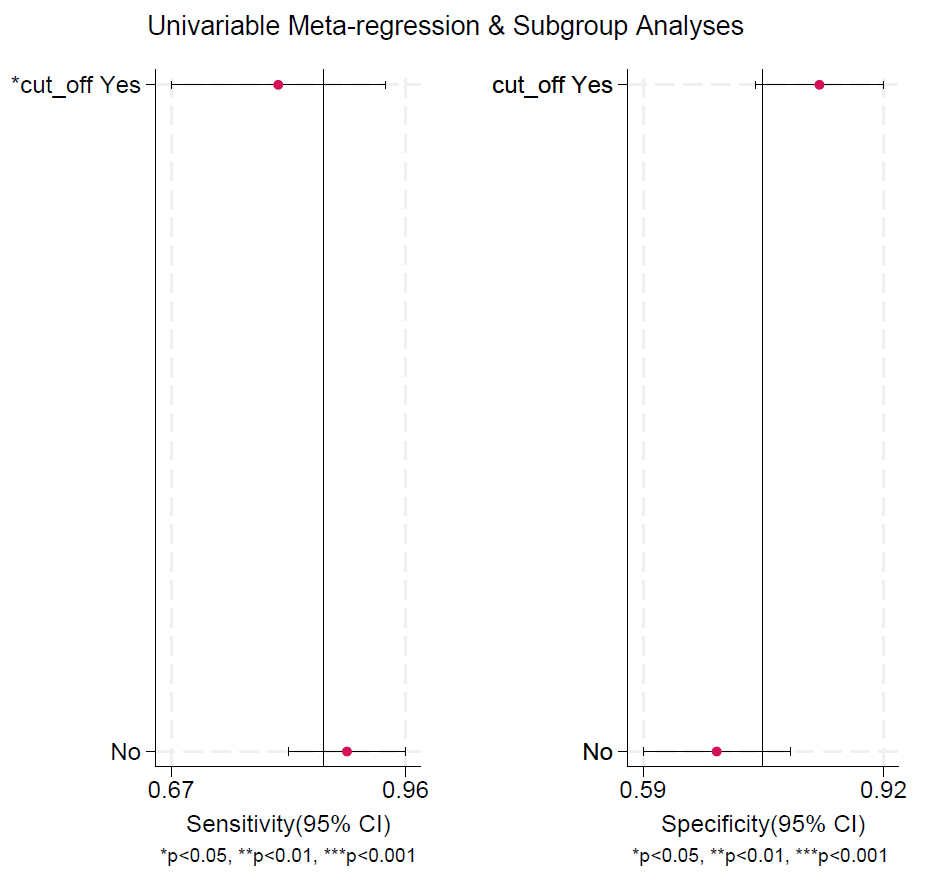


Supplementary Figure 5: Univariable Meta-regression and Subgroup Analysis of Diagnostic Sensitivity and Specificity by Patient Selection in LUS Studies


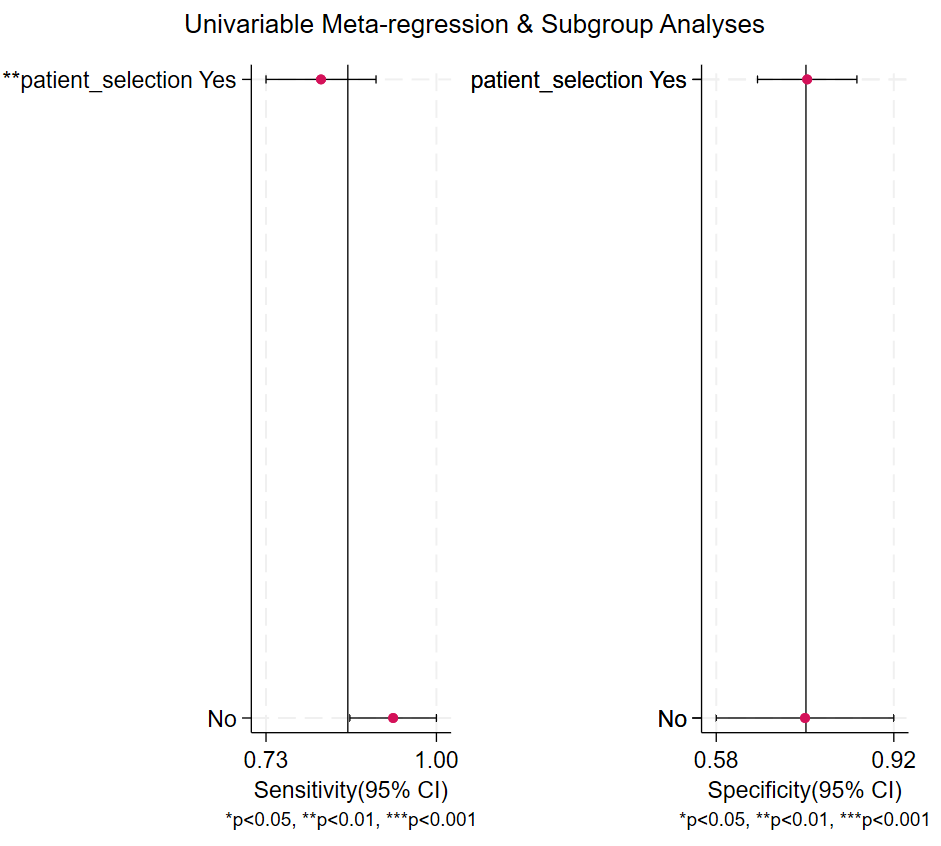


Supplementary Figure 6: Univariable Meta-regression and Subgroup Analysis of Diagnostic Sensitivity and Specificity by Study Quality in LUS Studies


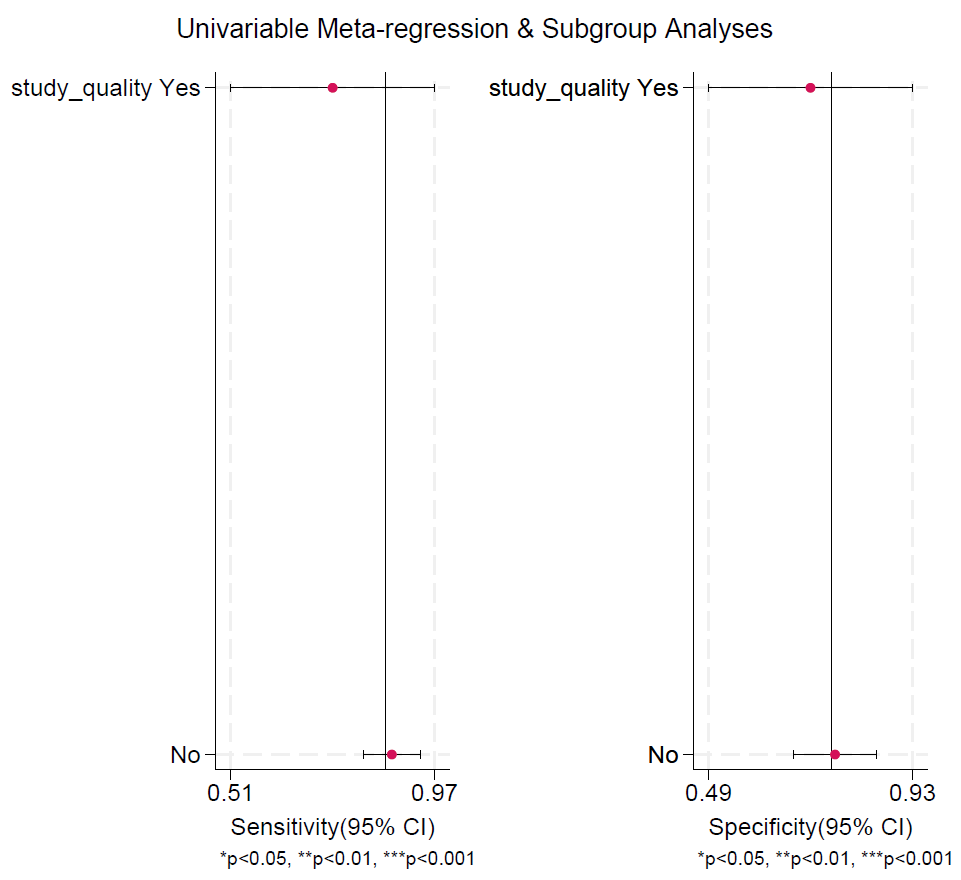


Supplementary Figure 7: Diagnostic and Influence Analyses for the Meta-analysis of LUS in Predicting Extubation Failure


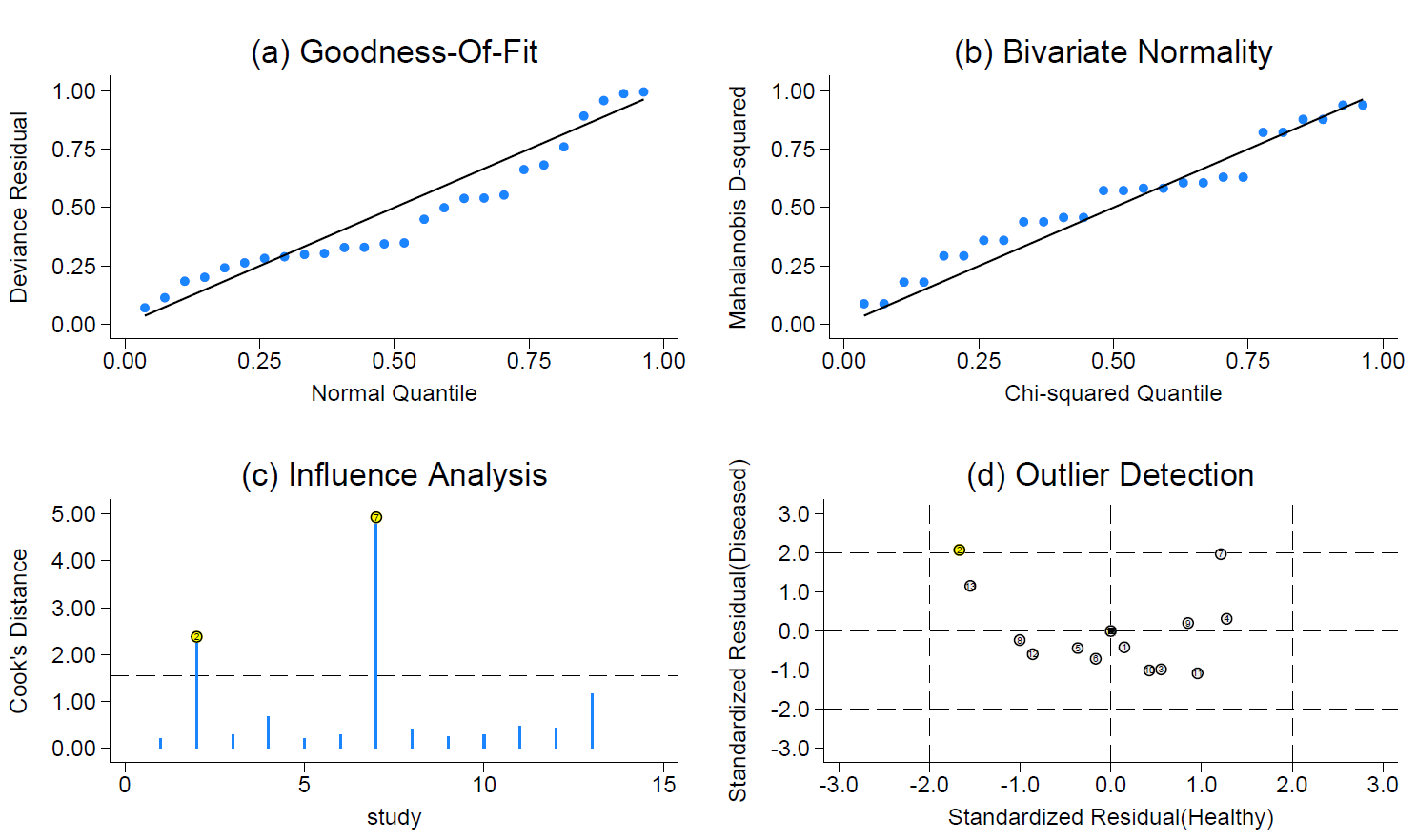


Supplementary Figure 8: Deeks' Funnel Plot Asymmetry Test for Publication Bias in LUS Studies Predicting Extubation Failure


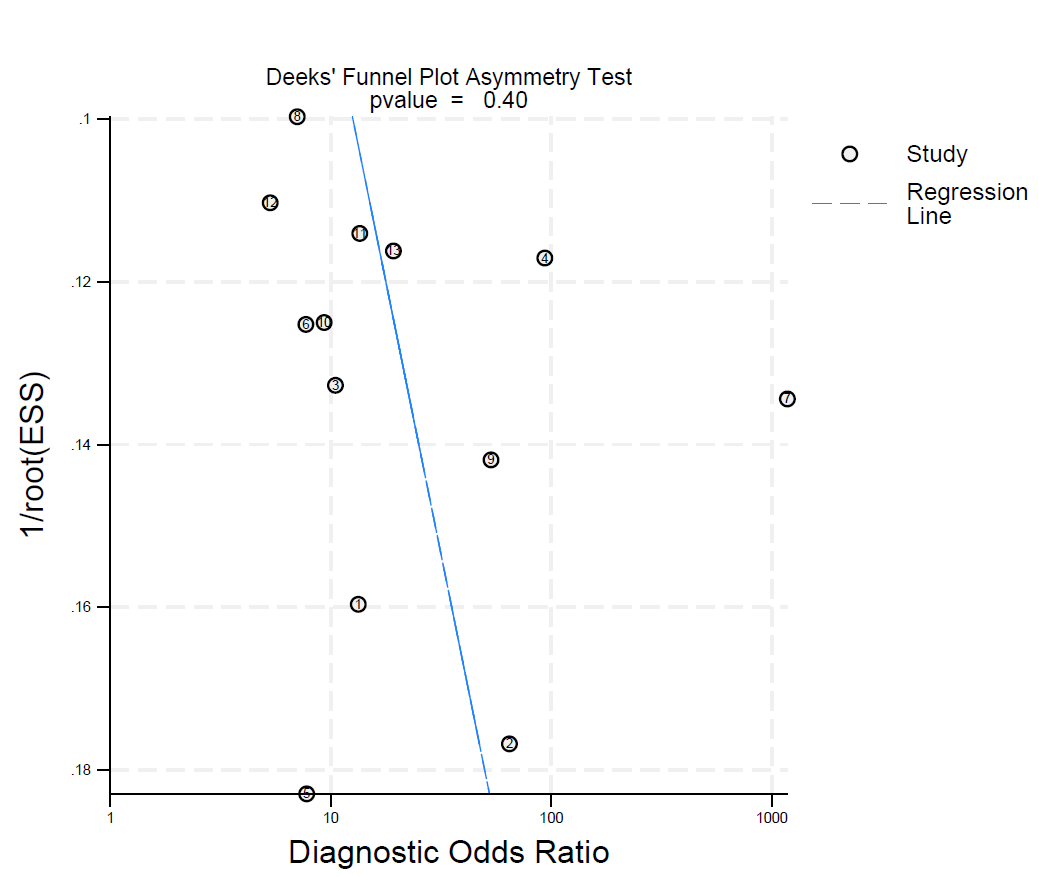

Supplement: Supplementary file 1 [file Data_Sheet_1.docx]
