# Supplementary material for: Diagnostic accuracy of lung ultrasound to predict weaning outcome: a systematic review and meta-analysis
Source: Front Med (Lausanne). 2024 Nov 1;11:1486636. doi: 10.3389/fmed.2024.1486636 (PMC11563988; doi:10.3389/fmed.2024.1486636)
Supplement: Supplementary file 3 [file Table_2.DOCX]

**Frontiers in medicine
Dear Editor,**

We are pleased to submit our manuscript entitled “Diagnostic accuracy of Lung Ultrasound to predict weaning outcome: A systematic review and meta-analysis” for consideration for publication as a major article in **Frontiers in medicine**. This study addresses a critical aspect of patient care in intensive care units (ICUs) – the optimization of timing for weaning from mechanical ventilation, which has significant implications for patient outcomes and healthcare resources.

Our systematic review and meta-analysis provide a comprehensive evaluation of the diagnostic accuracy of lung ultrasound (LUS) in predicting weaning failure in mechanically ventilated patients. By rigorously synthesizing data from 14 studies, encompassing 988 patients, our analysis highlights the potential of LUS as a reliable predictive tool for extubation outcomes. The nuanced findings from our meta-regression, exploring factors like LUS thresholds and study biases, offer valuable insights into enhancing the clinical utility of LUS in ICU settings.

We believe that our findings contribute significantly to the existing literature by quantifying the sensitivity and specificity of LUS in a clinical context, thereby providing a robust evidence base to guide clinical decisions regarding mechanical ventilation. Given the clinical importance of accurately predicting weaning outcomes to prevent complications associated with premature or delayed extubation, our study holds substantial relevance for your readership.

This work is original and has not been published elsewhere, nor is it currently under consideration by another journal. All authors have contributed significantly to the research and preparation of the manuscript and have approved its final version for submission. We have no conflicts of interest to declare.

We appreciate your consideration of our work and look forward to the possibility of contributing to **Frontiers in medicine**. Please do not hesitate to contact us should there be any questions regarding our submission.

Thank you and best regards,

Sincerely yours,

**Corresponding author**:

**Heling Zhao**, M.D., Ph.D., Department of Critical Care Medicine, Hebei Medical University, Shijiazhuang City, 050000,China. Department of Intensive Care Unit, Hebei General Hospital, Shijiazhuang City,050000, China. hlzhbgh@163.com.
